# Continuity of Vaccination Across the Life Course: Poland in Comparison with Selected EU/EEA Countries and the United Kingdom

**DOI:** 10.3390/vaccines14090802

**Published:** 2026-09-11

**Authors:** Ewa Benedyk-Lorens, Maria Leśniak, Olga Lorens, Jadwiga Wójkowska-Mach

**Affiliations:** 1 Department of Medicine and Health Sciences, Faculty of Medicine, University of Applied Sciences in Tarnow, 33-100 Tarnow, Poland; jadwiga.wojkowska-mach@uj.edu.pl; 2 Department of Internal Medicine and Nephrology with Dialysis Centre, St. Luke’s Provincial Hospital, 33-100 Tarnow, Poland; marialesniak68@gmail.com; 3 Independent Researcher, Kraków 30-798, Poland; 13olgalo13@gmail.com; 4 Department of Infection Control and Mycology, Chair of Microbiology, Jagiellonian University Medical College, 31-501 Krakow, Poland

**Keywords:** vaccination, vaccination schedule, mandatory vaccination, recommended vaccination, vaccination coverage, preventive healthcare

## Abstract

**Background/Objectives:** Vaccination represents one of the most significant achievements of modern medicine and public health. However, the scope, mandatory status, and vaccination coverage vary considerably across European countries. **Methods:** This narrative review compares selected elements of vaccination programmes in Poland, purposively selected EU/EEA countries, and the United Kingdom. Official data from the WHO, UNICEF, ECDC, EMA, and national sources were used to delineate vaccination policies, reimbursement models, delivery arrangements, coverage rates, and educational approaches. Due to temporal and methodological heterogeneity, the findings were interpreted descriptively. **Results:** Poland, together with Latvia, has the largest number of vaccinations classified as mandatory among the EU/EEA countries included in this comparison. Vaccination is mandatory in only nine EU/EEA countries; however, several countries without such policies also report high vaccination coverage in settings characterized by extensive public education and accessible vaccination services. Adult vaccination policies, recommendations, and reimbursement frameworks for influenza, pneumococcal, and RSV vaccines vary significantly depending on the country, target population, age criteria, and scope of funding. **Conclusions:** This comparison indicates a potential gap between the structured delivery of childhood vaccinations and the predominantly recommendation-based adult vaccination approach in Poland. Strategies utilized in selected European countries suggest that life-course vaccination continuity can be enhanced through accessible delivery systems, adequate reimbursement, systematic assessment of vaccination status, active engagement of healthcare professionals, sustained public communication, and trust-building. Mandatory status alone should not be regarded as sufficient to ensure high vaccination coverage.

## 1. Introduction

Vaccination is a vital component of the prevention of infectious diseases, particularly in older adults with chronic comorbidities, significantly reducing the risk of severe clinical course [1]. It represents one of the most significant achievements of modern medicine and public health [2]. A little over a century ago, pneumonia, diarrheal diseases, and infectious diseases such as tuberculosis, dysentery, and diphtheria were the leading causes of mortality globally and in Poland [3,4,5]. The advancement of medicine, particularly bacteriology and vaccinology, alongside the discovery of antibiotics, eliminated diseases that were previously widespread. The 20th century witnessed a substantial decline in infectious disease mortality rates; for instance, in the United States mortality rate decreased from 797 deaths per 100,000 population in 1900 to 59 deaths per 100,000 population in 1996 [4].

The origins of vaccinology date back to the late 18th century when the English physician Edward Jenner observed that individuals who contracted the relatively mild cowpox developed immunity to deadly smallpox. In 1796, he conducted an experiment involving inoculation of 8-year-old James Phipps with material collected from a fresh cowpox lesion from a milkmaid, Sarah Nelms. The method proved effective; following subsequent exposure to material from a human smallpox sore, the boy did not contract the disease [6,7]. Since the history of vaccination in most countries began with smallpox inoculation, it is widely accepted that these procedures marked the inception of vaccinology; the word “vaccine” itself derives from the Latin word “vacca”, meaning cow [8]. After Jenner’s success, it took nearly a century to develop the next vaccine, this time against rabies, introduced by Louis Pasteur in 1885 [9], after which subsequent vaccines steadily emerged. Poland has also made a significant contribution to the development of vaccinology. Rudolf Weigl, a Polish professor who worked at the Faculty of Biology of the Jan Kazimierz University in Lvov starting in 1920, conducted research on the production and introduction of epidemic typhus vaccines; his labour-intensive production technology was eventually superseded by a more scalable method developed for mass production in the United States [10,11].

In the modern era of genetic engineering, advanced and highly sophisticated vaccines have been developed to prevent more than 30 infectious diseases, saving several million lives annually [12]. Currently, the concept of vaccinology has been entirely redefined; contemporary formulations are no longer based solely on attenuated pathogens but are products of advanced technologies, including mRNA-based platforms. These present a satisfactory safety profile alongside steadily improving efficacy, thereby facilitating further development in this medical field [13].

Following the success of the global smallpox eradication programme [14], in May 1974 the World Health Organisation (WHO) established the Expanded Programme on Immunisation (EPI), signalling a proactive commitment to expanding the protective benefits of vaccination. In its current form, EPI encompasses protection against numerous global and regional pathogens across all age groups. The implementation of life-course vaccination continuity within this framework is determined by national policy decisions [15,16]. In Poland, the scope and availability of vaccinations are regulated by a legal act known as the Vaccination Schedule (Program Szczepień Ochronnych [abbreviated PSO], commonly referred to as Kalendarz Szczepień [Vaccination Calendar]). This document outlines the current list of fully reimbursed mandatory vaccinations, as well as partially reimbursed or non-reimbursed recommended vaccinations [17]. In most European countries, vaccinations are not mandatory; however, these countries annually update guidelines on recommended preventive vaccinations, issuing corresponding documents such as the Routine Immunisation Schedule in the UK, the Impfkalender in Germany, and the Österreichischer Impfplan in Austria. In eight countries (Greece, Spain, France, Germany, Italy, Lithuania, the Netherlands, and Denmark), vaccines are reimbursed despite the absence of a legal mandate [18,19,20,21,22,23,24,25,26,27,28].

Vaccination programmes have traditionally focused on children and adolescents, resulting in well-established systems for delivering and monitoring routine childhood vaccination. However, protection against vaccine-preventable diseases is necessary throughout the life course. Once individuals leave organised childhood vaccination programmes, responsibility for initiating and completing recommended vaccinations becomes less clearly defined and frequently depends on patient awareness, recommendations from healthcare professionals, accessibility, and reimbursement. This gap is particularly critical in older adults and individuals with chronic conditions, who are at an increased risk of severe outcomes from vaccine-preventable diseases but may remain insufficiently protected.

The aim of this narrative review is to compare selected elements of national vaccination programmes in Poland, EU/EEA countries, and the United Kingdom, with particular attention to the transition from mandatory childhood vaccination programmes to recommended vaccinations in adulthood. The review examines differences in vaccination policies, reimbursement, vaccine delivery, and public education, ultimately identifying strategies that may support continuity of vaccination across the life course and improve adult vaccination uptake in Poland.

## 2. Materials and Methods

### 2.1. Review Design and Geographical Scope

This narrative review was designed to compare selected elements of national vaccination programmes in Poland, purposively selected European Union and European Economic Area (EU/EEA) countries, and the United Kingdom. Poland served as the primary country of interest. The geographical framework was restricted to the EU/EEA and the United Kingdom, because these nations operate within a shared European context of public health cooperation, regulatory oversight, professional mobility, and international reporting, while retaining individual national sovereignty over vaccination policies, financing, and delivery.

Comparator countries were selected purposively rather than through statistical sampling. Selection criteria included availability, completeness, accessibility, and verifiability of official data regarding national vaccination schedules, recommendations, eligibility criteria, reimbursement frameworks, vaccine delivery systems, educational initiatives, and vaccination coverage rates. Additionally, the selection process aimed to capture countries with diverse policy frameworks and varying levels of reported vaccination coverage, thereby minimizing selection bias toward exclusively high-performing vaccination systems. Consequently, the selected countries were analysed as policy-relevant case examples rather than a statistically representative sample of the broader European region.

### 2.2. Data Sources and Source Hierarchy

Data were obtained from reports, databases, and official documents published by the World Health Organisation (WHO), the United Nations Children’s Fund (UNICEF), the European Centre for Disease Prevention and Control (ECDC), the European Medicines Agency (EMA), and national public health or governmental authorities.

The sources served complementary purposes and were not considered interchangeable. Harmonized WHO/UNICEF estimates and ECDC data were preferentially utilized for vaccination coverage and epidemiological indicators. EMA documents were consulted for regulatory information, including vaccine authorisation and approved indications. National sources were used primarily to identify current vaccination schedules, mandatory and recommended vaccinations, target populations and risk groups, eligibility criteria, reimbursement frameworks, modes of vaccine delivery, and recently introduced or announced policy changes. Where discrepancies were identified, preference was given to the most recent official source directly responsible for the relevant indicator or policy. National public health or governmental sources were prioritized for current country-specific policies, whereas harmonized international sources were preferred for quantitative cross-country comparisons.

### 2.3. Data Extraction and Classification

For each quantitative indicator, the following variables were extracted where available: the reference year or reporting period, target population, indicator definition, numerator and denominator, reporting unit, and data source. For policy-related data, the extracted variables included the legal status of vaccination, target populations and risk groups, eligibility criteria, reimbursement, vaccine delivery arrangements, and the professional groups authorised to administer vaccines. Official institutional websites and bibliographic databases were searched using combinations of terms related to vaccination schedules, mandatory vaccination, adult vaccination, vaccine reimbursement, vaccination coverage, vaccine delivery, and public education. Reference lists of relevant reports and publications were also screened. The extracted information was classified into four categories:

- current vaccination policies and recommendations;
- forthcoming policy changes;
- vaccination coverage and programme implementation indicators;
- epidemiological data, including historical context used to provide disease-specific backgrounds.

Data on the availability, recommendation, or reimbursement of a vaccine were not interpreted as evidence of vaccine uptake or national programme effectiveness. Similarly, epidemiological indicators and vaccination coverage estimates were not assumed to represent the effects of current policies unless their temporal sequence and methodological comparability supported such an interpretation. Data were extracted by E.B.-L. and O.L. and independently verified by M.L. Discrepancies were resolved by a third reviewer (J.W.-M.).

### 2.4. Temporal Framework

The literature and policy search was last updated on 29 August 2026. National policies active on that date were classified as current. Announced changes scheduled to take effect after that date were classified as forthcoming policies.

For vaccination coverage and epidemiological indicators, the most recent data available as of the cut-off date were extracted. The corresponding reference year or reporting period was recorded for each indicator. When comparable estimates for a common year were unavailable, data from different periods were retained solely to provide a descriptive overview. These estimates were not treated as a single cross-sectional dataset and were not used to infer the effects of current vaccination policies.

Historical epidemiological data were included only when they provided relevant context for the development or interpretation of vaccination strategies. Such data were clearly distinguished from current policy indicators and measures of contemporary programme implementation.

### 2.5. Assessment of Data Comparability

The included sources were developed for different surveillance, regulatory, and policy purposes and were therefore not assumed to be methodologically equivalent. Differences in reporting periods, target populations, population denominators, data collection methods, indicator definitions, reporting units, and data completeness were accounted for when interpreting the findings.

Where indicators were based on different definitions, populations, or reference periods, the results were presented descriptively and were not interpreted as precise measures of inter-country discrepancies. Direct quantitative comparisons were reserved for indicators derived from sufficiently homogenous reporting frameworks, definitions, and reference periods. Accordingly, the findings were interpreted as a descriptive comparison of selected national approaches rather than as an estimate of the overall association between vaccination policies and coverage across Europe, or as a standardized ranking of national vaccination programmes.

### 2.6. Determination of the DTP1-to-DTP3 Dropout Rate

The dropout rate for the diphtheria, tetanus, and pertussis vaccine series was used as an indicator of completion of vaccination series in children. It was calculated as follows:

$$DTP1\;to\;DTP3\;dropout\;rate\;\left(\%\right)\;=\;\frac{DTP1\;percentage-DTP3\;percentage}{DTP1\;percentage}\times 100$$

The indicator represents the relative proportion of children who received the first dose of a DTP-containing vaccine but did not receive the third dose. It is distinct from the absolute percentage-point difference between DTP1 and DTP3 coverage. UNICEF similarly defines the DTP1-to-DTP3 dropout rate as the proportion of children who started the DTP series but did not complete it. When aggregate coverage estimates are used rather than individual vaccination records, the dropout rate is interpreted as a population-level indicator of programme continuity and not as an individual-level estimate of vaccination-series completion. Aggregate data do not confirm that the individuals included in the DTP1 and DTP3 estimates represent the same paediatric cohort [29].

### 2.7. Analytical Approach and Interpretation

The analysis was descriptive. The differences between individual countries were summarized with respect to vaccination policies, reimbursement frameworks, programme delivery arrangements, educational initiatives, vaccination coverages, and selected epidemiological indicators. Given the narrative design of the review, the purposive selection of comparator countries, and the methodological and temporal heterogeneity of the included sources, the analysis was not intended to estimate the causal effects of mandatory vaccination, reimbursement policies, delivery arrangements, or educational interventions on vaccination coverage. Expressions implying causality were therefore avoided unless supported by evidence from longitudinal, comparative, or interventional studies. The observed differences were described using non-causal formulations, including “associated with,” “coexisted with,” “may support,” and “may contribute to.” Throughout the manuscript, the term “vaccination” was used when referring to vaccine administration, vaccination schedules, programmes, policies, uptake, and coverage. The term “immunisation” was retained solely in the official names of programmes, databases, or documents, or when referring more broadly to the biological process of acquiring immunity to a disease.

## 3. Vaccination in Poland

The Duchy of Warsaw (1807–1815 years), a semi-autonomous Polish state established by Napoleon Bonaparte primarily from former Prussian-annexed territories, was the first polity in the Polish lands to introduce compulsory smallpox vaccination. Smallpox vaccination was also practiced in the Polish territories under Prussian rule by the mid-nineteenth century; more broadly, in Prussia, vaccination had already become a matter of state responsibility before the 1870s, and these policies were later consolidated in the German Empire’s compulsory vaccination law of 1874 [30]. Currently, Poland mandates nine fully funded, compulsory vaccinations, making it a leader among European countries. Non-compliance with mandatory childhood vaccination in Poland may result in administrative enforcement proceedings, including repeated fines imposed to compel compliance. Additional consequences may arise in individual legal proceedings, while vaccination status may also be considered in the admission criteria adopted by some local childcare institutions.

The list of recommended (non-mandatory) vaccinations in Poland encompasses vaccines protecting against 26 infectious diseases, with specific recommendations depending on the patient’s age, comorbidities, occupation, place of residence (applicable to residential care and treatment facilities), contact with young children, or planned travel [17].

Reimbursement for recommended vaccinations is primarily targeted at older adults and individuals with chronic comorbidities. Among the recommended vaccinations for individuals aged over 65 years, vaccines against influenza, RSV (Respiratory Syncytial Virus)*,* and herpes zoster (shingles) are provided free of charge. Conversely, individuals aged 61–64 years receive 50% reimbursement for the RSV vaccine. The herpes zoster vaccination for adults is reimbursed at 50%, provided there is a coexisting increased risk of contracting shingles. In the case of pneumococcal vaccinations, individuals aged over 65 years can receive the vaccine free of charge, provided they have a coexisting increased risk of invasive pneumococcal disease [31].

Unfortunately, for individuals who have not yet reached 65 years of age, the cost of vaccines against RSV or herpes zoster remains high; even with 50% reimbursement, it still constitutes approximately one-fifth of the minimum retirement pension.

In addition to the mandatory vaccinations derived from the Vaccination Schedule, children in Poland can receive certain influenza vaccines free of charge, and from the age of 9 years they can be vaccinated free of charge against HPV types 16 and 18 [31]. In Poland, HPV vaccination is currently recommended and publicly funded for eligible children and adolescents. A regulatory change has been announced under which HPV vaccination is expected to be incorporated into the mandatory childhood vaccination programme from 1 January 2027. As this change was not yet in force by the review cut-off date, it is presented here as a forthcoming policy rather than as a component of the current vaccination schedule. Its association with vaccination uptake, acceptance, and programme completion cannot yet be evaluated.

Pregnant women receive targeted care and can be vaccinated free of charge against pertussis (whooping cough), influenza, and RSV.

In Poland, an adult patient can be vaccinated by a physician, dentist, a trained medical assistant (felczer), nurse, midwife, school hygienist, or paramedic, as well as a physiotherapist, laboratory diagnostician, or pharmacist; furthermore, vaccines can also be administered directly at a pharmacy [31,32,33].

The range of healthcare professionals authorised to administer vaccines continues to grow. Pharmacists gained this authority in 2021, though it was initially restricted to non-reimbursed influenza vaccines. This scope was broadened on 14 February 2025, permitting pharmacists to issue prescriptions and administer reimbursed and non-reimbursed vaccines against 18 infectious diseases [34].

The relationship between mandatory vaccination policies, vaccine acceptance, and vaccination coverage is complex and depends on the broader legal, organisational, and social contexts. Regrettably, the number of children who are not vaccinated or only partially vaccinated is rising in Poland, particularly in the eastern voivodeships [35].

The DTP1-to-DTP3 dropout rate, which represents the relative proportion of children who initiated but did not complete the three-dose series, increased in Poland in 2024 compared with 2023 [35,36]. Furthermore, the number of individuals who have received recommended but non-mandatory vaccines remains low (Table 1).

To increase vaccination coverage and mitigate vaccination discontinuation, various public health campaigns are underway. Flagship initiatives include “Inoculate the Will to Vaccinate” (“Zaszczep w sobie chęć szczepienia”), initiated in November 2013, “Vaccinations Protect–Trust Science” (“Szczepienia Chronią-Zaufaj Nauce”), which served as the official theme and slogan of the Polish edition of European Immunisation Week in 2025 and 2026. Next, the “Med-Fake” project was launched in 2021. These educational and scientific initiatives aim to combat medical misinformation and encourage vaccination uptake. Furthermore, these campaigns are based on evidence-based medicine principles, carried out in collaboration with academic institutions, and continuously adapted to legislative changes [37,38,39,40].

Regrettably, public health programmes in Poland frequently encounter insufficient public engagement. A prime example is oncological prevention, where participation remains inadequate despite steadily increasing financial investment. For instance, the participation rates for the breast cancer prevention screening programme conducted in Poland from 2012 to 2015 were between 20% and 40%, whereas they ranged from 50% to 80% in most European countries [41].

## 4. Vaccinations in Countries of the European Union (EU), the European Economic Area (EEA) and the United Kingdom

According to the WHO, vaccinations prevent between two and three million deaths worldwide each year. It is assumed that expanding access to vaccines could prevent an additional 1.5 million deaths [12]. Nevertheless, efficacy and, more notably, safety of vaccinations continue to be questioned in many regions of the world [42].

The WHO/UNICEF Estimates of National Immunisation Coverage (WUENIC) annually publishes estimated data on global vaccination coverage based on reports prepared by member states (WHO, UNICEF) [43]. According to a 2024 UNICEF report, Europe along with Central Asia had 93% vaccination coverage rate for DTP3, which is recognized as a key indicator of vaccination system performance, because the DTP vaccine requires three separate contacts with the healthcare system [44].

In the European Union, vaccination policy and the organisation of vaccination programmes remain primarily within the competence of individual Member States, while the European Commission supports coordination, information exchange, and common strategic action across countries. Within this framework, the EMA serves a regulatory rather than a programmatic role: it conducts the scientific evaluation of vaccines for quality, safety, and efficacy, after which the European Commission may grant a centralized marketing authorisation valid throughout the EU. Following authorisation, the EMA and the EU regulatory network remain responsible for post-authorisation pharmacovigilance and benefit–risk assessment, while vaccine monitoring in the EU/EEA is further supported through collaboration with the ECDC [45,46,47,48].

Similar to the regulatory shifts in Poland, the integration of pharmacy-based vaccination services by community pharmacists has been expanding rapidly across Europe. Currently, pharmacists are authorised to administer influenza and COVID-19 vaccines in 15 European countries, including Belgium, Germany, Latvia, Luxembourg, Romania, and the United Kingdom. In a subset of nine nations, namely Denmark, France, Greece, Ireland, Italy, Norway, Portugal, Switzerland, and the United Kingdom, this authority extends to many other vaccines, including pneumococcal, meningococcal, tick-borne encephalitis (TBE), hepatitis A and B, HPV, rotavirus, varicella, and shingles vaccines. Furthermore, they provide travel-specific vaccinations (e.g., cholera, typhoid fever, and Japanese encephalitis) and administer urgent vaccinations or post-exposure prophylaxis against rabies, tetanus, diphtheria, and pertussis, including serum administration. Alternatively, models applied in Croatia, Estonia, and the Netherlands use pharmacies as clinical vaccination centres where vaccines are administered on-site by visiting physicians or nurses [49].

Vaccination coverage across European countries is associated with multiple epidemiological, organizational, financial, and sociocultural factors, including public communication and educational activities [50,51]. Countries also differ significantly in terms of their vaccination budgets, national policies (including the list of free mandatory vaccinations and reimbursement of recommended vaccines), healthcare accessibility, and public health education [51,52].

Across the European Union, national vaccination schedules differ regarding the age at which vaccines are administered, the number and spacing of doses, the use of combination versus single-antigen products, the target groups covered, and whether selected vaccinations are mandatory or recommended; these differences reflect variation in disease burden, health system organisation, resources, and policy choices. At the same time, EU/EEA schedules are broadly similar in that all countries maintain structured national programmes and include a common core of childhood vaccines against diseases such as measles, mumps, rubella, diphtheria, tetanus, pertussis, poliomyelitis, and *Haemophilus influenzae* type b, with seasonal influenza vaccination recommended for key risk groups across all countries [52].

Nine EU/EEA countries have mandatory multi-antigen childhood vaccination schedules, while Belgium mandates vaccination against poliomyelitis only (Table 2). Poland and Latvia have the highest number of mandatory, free vaccinations. While vaccination against DTP, polio, Hib, and MMR is mandatory in all EU/EEA countries that implement compulsory vaccination, the distribution of the remaining vaccines varies [52].

In countries without mandatory vaccination policies, vaccination coverage and the DTP dropout rates vary (Table 3) [36].

Among the EU/EEA countries that do not implement mandatory vaccinations, Estonia and Romania demonstrate the lowest coverage rates. Specifically, Estonia and Romania had the lowest DTP1 coverage among the countries included in Table 3, at 82% and 85%, respectively. DTP3 coverage was 81% in Estonia and 66% in Romania, with the DTP1-to-DTP3 dropout rate being particularly high in Romania at 22.4% [36]. In the remaining EU/EEA countries without mandatory vaccinations, DTP coverage is over 90% (Table 4).

In countries without mandatory vaccination that achieve high vaccination coverage, numerous national public health campaigns are conducted. Notable examples include Germany’s “Zusammen geschützt” (Together Protected) initiative, as well as Belgium’s seasonal campaign launched under the French title “Campagne de vaccination automne/hiver” and the Dutch title “Herfst-en wintervaccinatiecampagne” (Autumn–Winter Vaccination Campaign). These national efforts are further augmented by widely promoted pan-European initiatives, such as the European Immunisation Week (EIW) and the #UnitedInProtection campaign. Populations in these countries frequently report highly positive attitudes toward preventive healthcare, viewing health as a core component of their quality of life [54,55].

HPV vaccination coverage provides one example of variation in the implementation of recommended vaccination programmes across Europe. Some countries report coverage exceeding 90%, including Iceland and Sweden, while several have reduced the coverage difference between girls and boys to below 10%. These values should, however, be interpreted in the context of differences in target populations, programme delivery, reporting periods, and data sources. These countries report higher HPV vaccination coverage than Poland; however, differences in data sources, reporting periods, programme delivery, and target populations limit direct comparison. In Poland, by 2025, HPV vaccination coverage reached only 28.92% among 15-year-olds and 33.62% among 14-year-olds [56].

Reimbursement systems vary by country but consistently aim to cover high-risk groups. For instance, in Belgium, the only mandatory vaccine provided free of charge to children is the polio vaccine; however, most childhood vaccinations are free, with only the rotavirus vaccine requiring out-of-pocket payment. Vaccinations for adults are either free of charge or reimbursed depending on the vaccine type, patient age, residence in long-term care facilities, or pregnancy status. The Superior Health Council (Conseil Supérieur de la Santé [CSS]/Hoge Gezondheidsraad [HGR]) updates the national vaccination schedule annually [52,57]. In Germany, vaccinations for both children and adults are provided free of charge, conditional upon being recommended by the Standing Committee on Vaccination (Ständige Impfkommission [STIKO]) [52,58].

Historical context from Jenner’s home country, the United Kingdom, is highly relevant, as it represents the birthplace of mandatory vaccination; the Vaccination Act of 1853 introduced the first universal mandate for infant smallpox vaccination (up to three months of age) in England and Wales. Parents who failed to vaccinate their children faced financial penalties or imprisonment [59]. Currently, the United Kingdom does not implement mandatory vaccinations; however, a structured schedule of recommended vaccination is maintained, and the state authorities actively supervise vaccination uptake. The COVER (Cover of Vaccination Evaluated Rapidly) epidemiological surveillance system is maintained to monitor vaccination coverage in children up to five years of age [60]. In the report for the first quarter of 2026, the DTP vaccination coverage among infants up to 12 months of age was 90.1% [61].

In the United Kingdom, numerous public health campaigns regarding vaccination are conducted, coordinated by the NHS (National Health Service) alongside the UKHSA (UK Health Security Agency). Within this framework, a campaign promoting influenza vaccination was conducted in 2025 among adults with chronic comorbidities. A prime example is the “Stay Strong, Get Vaccinated” educational programme, which promoted vaccination among patients with chronic kidney disease, diabetes, chronic respiratory diseases, neurological conditions, and immunosuppression [62]. The UKHSA also operates the international “e-Bug” programme, which provides educational tools for implementation in primary and secondary schools. This initiative, which has also been adopted in Poland, targets children, teachers, and parents [63]. Furthermore, British primary care physicians (GPs—General Practitioners) receive support through dedicated educational materials [64]. School-based health education may contribute to higher vaccination awareness and may potentially foster long-term vaccine uptake later in life.

Following implementation of the national HPV vaccination programme, reductions in cervical cancer and CIN3 incidence were observed among eligible birth cohorts in England. By 30 June 2019, an estimated 448 fewer cervical cancer cases and 17,235 fewer CIN3 cases than expected had occurred [65].

## 5. Overview of Selected Vaccinations Against Airborne Diseases for the Adult Population in the EU

### 5.1. Influenza

The ECDC estimates that seasonal influenza causes up to 50 million symptomatic infections annually in the EU [66]. Seasonal influenza imposes a substantial burden of morbidity and mortality in Europe, particularly among individuals aged 65 years and older as well as those with comorbidities [67]. Notably, influenza-associated mortality is significantly higher among individuals aged over 65 years compared to younger populations [53,68]. The ECDC projected that vaccination programmes in EU countries during the 2024–2025 season could prevent between one-quarter and one-half of all influenza-related hospitalisations among adults aged 65 years and older [69,70].

Influenza vaccinations are classified as recommended seasonal vaccinations. All influenza vaccines require annual updates, as post-vaccination immunity remains relatively short-lived, and vaccine composition must be antigenically matched to currently circulating virus strains [71]. In Europe, seasonal influenza vaccination is generally undertaken before the onset of increased influenza activity, although vaccination may continue during the season in accordance with national recommendations [72]. Specific indications for influenza vaccination within the EU include risk-factor-based vaccination tailored to individual clinical profiles (e.g., pregnancy, lifestyle, health status, comorbidities), occupational vaccination (e.g., healthcare workers), and routine age-based vaccination. The recommended age for initiating vaccination in older adults varies by country and predominantly ranges from 60 years (e.g., Germany, Greece, Hungary, Ireland) to 65 years (e.g., Cyprus, Czechia, Denmark, France) [73].

Currently, three types of vaccines are available: inactivated influenza vaccines (IIVs), live attenuated influenza vaccines (LAIVs), and recombinant hemagglutinin (HA) vaccines [74]. Formulations containing the inactivated virus are characterized by a high safety profile and relatively low production costs [75]. In the 2025/2026 season, virtually all vaccines available for adults in the EU are inactivated formulations containing two influenza A strains and either one (trivalent, implemented in 13 out of 27 member states) or two (quadrivalent, implemented in 7 out of 27 member states) influenza B strains. The following influenza vaccines were granted central marketing authorisations by the EMA: Fluad Tetra™, Fluenz Tetra™, Flucelvax Tetra™, and Supemtek™ [76]. Furthermore, there are seasonal influenza vaccines authorised at the national level within individual European countries (Table 5) [73].

### 5.2. Streptococcus Pneumoniae

Pneumococcal infections remain an important health challenge in Europe, particularly among older adults and individuals with underlying conditions [97,107]. According to the ECDC report, a total of 17,700 confirmed cases of IPD were recorded in European countries in 2022, with the highest incidences reported in France, Spain, and Poland. Concurrently, it is estimated that the IPD notification rate in the EU was approximately 5 cases per 100,000 population, though these rates are lower in certain southern and eastern parts of Europe. Among cases with a known outcome, mortality was highest among adults aged 65 years and older, with a case fatality rate of 17.1% [108].

Pneumococci include approximately 100 serotypes, and associated vaccines differ primarily in terms of their valency. The vaccines utilized in the EU can be divided into two groups: pneumococcal conjugate vaccines (PCVs), administered as a single-dose schedule, which include 13-(Prevenar 13), 15-(Vaxneuvance), 20-(Prevenar 20), and 21-valent (Capvaxive) formulations; and pneumococcal polysaccharide vaccines (PPSVs), also administered as a single-dose schedule, which include 23-valent formulations (Pneumovax 23), adult pneumococcal vaccination schedules vary according to age, risk status, previous vaccination history, and national recommendations [109,110,111,112,113]. In many Western European countries, vaccines with a valency of 13 or higher are widely used, and most countries are intensively transitioning to the latest 15-valent and 20-valent formulations. The increased utilization of multivalent vaccines is medically justified, as in 2022, within the group of adults aged 65 years and older, approximately 71% of IPD cases were caused by serotypes included in the PPSV23 vaccine, and 41% by one of the serotypes found in the 13-valent PCV13 vaccine [108]. Unfortunately, in Poland, depending on the patient group, only 10- and 13-valent pneumococcal vaccines are reimbursed.

Furthermore, vaccination coverage among the adult population in Europe varies considerably by country and age group. A 2021 report indicated that the estimated percentage of individuals vaccinated against pneumococci in EU countries is highly diverse, ranging from 18% to 88% depending on the population group. Conversely, among individuals in clinical risk groups and senior citizens, the overall vaccination coverage is substantially lower, standing at only around 20–30% [114].

In Europe, recommendations for pneumococcal vaccination differ, with stark discrepancies between Eastern and Western Europe. Pneumococcal vaccination is recommended for the elderly in all EU countries except Estonia [115]. Sixty percent of countries recommend pneumococcal vaccination for children and individuals with specific risk factors (e.g., lifestyle, health status, comorbidities), alongside routine age-based vaccination for the elderly. However, individual countries differ in their definitions of risk groups and their establishing of age criteria (Table 6) [115].

### 5.3. Respiratory Syncytial Virus (RSV)

RSV is a major cause of acute lower respiratory tract infections in young children and a leading contributor to severe respiratory disease among older adults and individuals with underlying conditions. Worldwide, approximately 100,000 deaths attributable to RSV are estimated to occur annually among children younger than five years, whereas the global mortality burden among adults remains less precisely characterized [125,126,127,128,129]. Furthermore, RSV frequently leads to hospitalisations among the elderly, particularly during the winter months [130]. Among adult residents of the European Union, RSV accounts for over 158,000 hospital admissions per year, with up to 92% of these involving individuals aged 65 years and older [131,132,133]. The average hospital stay is longer for adult patients with RSV infection than for those with influenza [134].

Current guidelines for older adults recommend the administration of a single vaccine dose at any time of the year; however, in regions where RSV exhibits seasonal patterns the maximum efficacy is achieved when the vaccine is administered immediately before the anticipated onset of the season [135]. Twelve out of twenty-seven EU member states recommend RSV vaccination. Seven countries recommend vaccination for pregnant women, whereas in Poland this vaccine is fully reimbursed for the eligible target population. Eight countries recommend vaccination for all adults aged ≥75 years, while Poland is the only nation that recommends vaccination from ≥60 years of age. In the case of individuals with risk factors, most countries recommend vaccination at a younger age, most commonly from ≥65 years. Risk factors may vary by country, but typically encompass chronic diseases, advanced age or frailty, immunosuppression, and metabolic syndromes [136].

Currently, three RSV vaccines were approved for use in adults by the US Food and Drug Administration (FDA) and the EMA. These include two protein subunit vaccines: Arexvy, an adjuvanted prefusion RSV F protein vaccine (adjuvanted RSVPreF3, GSK), and Abrysvo, a bivalent prefusion RSV F protein vaccine (RSVpreF, Pfizer), alongside one mRNA-based vaccine mRESVIA (mRNA-1345, Moderna) [137]. In early 2026, the indications for the adjuvanted RSVPreF3 vaccine in the EU were expanded to include all adults aged 18 years and older, thus bringing the eligibility criteria for Arexvy into alignment with those for Abrysvo and mRESVIA [138,139,140]. US recommendations advise administration before the anticipated RSV season in regions with seasonal circulation; national European guidance may differ.

## 6. Implications for Strengthening Life-Course Vaccination in Poland

Healthcare professionals play a key role in identifying vaccination needs and discussing vaccination with patients throughout their life course. Their training should extend beyond the provision of occasional educational materials and should include extensive vaccination-related competencies. Those should be embedded in the undergraduate, postgraduate and specialty medical education, as well as the continuing education of medical professionals. Such training should cover current vaccination schedules, indications for specific age and risk groups, contraindications, vaccine safety, assessment of vaccination status, and practical aspects of vaccine delivery. It should also develop communication skills required to address patients’ concerns, respond to misinformation, and support informed decision-making. Regularly updated guidance and educational materials, such as those provided to GPs in the United Kingdom, may complement structured professional education but should not replace it. Improving these competencies is particularly relevant in Poland, where adult vaccination is predominantly recommendation-based and therefore depends substantially on healthcare professionals proactively recognizing vaccination needs and initiating discussions with patients.

Public trust in vaccines, healthcare professionals, and public institutions is critical to the successful implementation of vaccination programmes. Low uptake does not alter the legal status of a mandatory vaccination but may indicate a gap between the mandate and its practical implementation. Mandatory status alone should therefore not be considered sufficient to ensure high vaccination coverage. Accessible services, clear responsibility for follow-up, professional recommendations, transparent communication, and proportionate enforcement are also necessary. Mandatory vaccination and trust-building should be viewed as complementary components of a comprehensive vaccination strategy rather than as mutually exclusive approaches. Poland’s planned inclusion of HPV vaccination in the mandatory childhood vaccination programme from 2027 illustrates the need to distinguish legal status from programme implementation. The introduction of a mandate should be accompanied by convenient school- and primary-care-based delivery, communication with parents and adolescents, healthcare professional engagement, monitoring of initiation and completion, and transparent evaluation of vaccination uptake and acceptance.

Norway introduced school-based HPV vaccination for 12-year-old girls in 2009. Reported coverage increased from 72.5% in 2009 to 87.3% in 2014 and subsequently approached 90%. Although these trends coincided with school-based delivery, the observational data do not allow the independent contribution of the delivery setting to be determined [141,142].

## 7. Conclusions

The comparison of Poland with selected EU/EEA countries and the United Kingdom highlights the importance of maintaining the continuity of vaccination across the life course. In Poland, vaccination during childhood and adolescence is delivered within a structured mandatory programme, whereas vaccination in adults is predominantly recommendation-based and depends more heavily on individual awareness, healthcare professional engagement, accessibility, and reimbursement. This transition may contribute to a fragmentation of responsibility for assessing vaccination status, recommending vaccines, and ensuring the completion of age- and risk-appropriate vaccination.

The reviewed European examples suggest several approaches that may be relevant to Poland. These include systematic assessment of vaccination status during routine healthcare encounters; a clearer assignment of responsibility for adult vaccination within primary and specialist care; proactive recommendations by healthcare professionals; a broader use of pharmacies, workplaces, schools, and other accessible vaccination settings where legally and organisationally appropriate; reminder and recall systems; consistent documentation of vaccination; and reduction in financial and administrative barriers. Sustained public education should complement these measures by addressing concerns transparently and strengthening trust in healthcare professionals and public institutions.

Mandatory vaccination should not be viewed as either sufficient to ensure high coverage or inherently counterproductive. Its legal status should be distinguished from enforcement, public acceptance, and actual uptake. Effective implementation requires accessible delivery, systematic follow-up, professional engagement, transparent communication, and sustained public trust. High coverage can be observed under both mandatory and voluntary systems, depending on the broader organisational, financial, and social context. Legal requirements should therefore be considered as a component of a comprehensive vaccination strategy rather than as a substitute for accessible services, professional recommendations, public communication, and trust.

Strengthening vaccination uptake in Poland requires a shift from a predominantly childhood-centred programme towards a coordinated life-course approach. However, because the included countries and data sources were selected purposively and differed with respect to reporting periods, definitions, and completeness, the findings should be interpreted as a descriptive comparison of policy approaches rather than as evidence of causal effects or as a representative ranking of European vaccination programmes. Further comparative and longitudinal research is needed to evaluate which combinations of policy, financing, delivery, and communication measures are most effective in the Polish context.

## 8. Limitations

This review has several limitations. First, the included international and national sources were heterogeneous with respect to data collection methods, reporting periods, target populations, population denominators, indicator definitions, and data completeness. Because vaccination coverage estimates were derived from disparate sources, including administrative records, registries, population surveys, and predictive models, they lacked methodological equivalence. Although current vaccination policies were assessed using a predefined cut-off date, the most recent available coverage and epidemiological data did not always correspond to the same reporting period. In addition, changes in vaccination recommendations, reimbursement, or delivery arrangements may precede the availability of data reflecting their implementation. Consequently, historical coverage estimates cannot be interpreted as outcomes of more recent policies. The purposive selection of countries and the descriptive, temporally heterogeneous nature of the evidence also preclude the causal attribution of differences in vaccination coverage to specific legal requirements, educational activities, reimbursement policies, or delivery models. Observed differences among countries may reflect multiple interacting legal, organisational, financial, cultural, and healthcare-system factors. The findings should therefore be interpreted as a descriptive comparison of selected vaccination policies and reported coverage patterns rather than as a contemporaneous, fully standardized inter-country ranking or a causal evaluation of national vaccination programme effectiveness.

Furthermore, the purposive selection of comparator countries may have introduced selection bias and consequently limits the generalizability of the findings to the entire EU/EEA region. Although countries were selected to illustrate variation in vaccination policies, delivery models, and reported coverage, the sample was not statistically representative. Specifically, countries with more complete and accessible official documentation were inherently more likely to be included. Consequently, the findings should be interpreted as a descriptive comparison of selected policy examples rather than as evidence of the average performance of European vaccination programmes.

## Figures and Tables

**Table 1:** Vaccination coverage in Poland in 2023 according to the WHO/UNICEF Estimates of National Immunisation Coverage (WUENIC) report and the National Institute of Public Health NIH—National Research Institute (NIPH NIH–NRI) report [35,36].

Disease/Pathogen	2023
* Tuberculosis	91%
* Hib	94%
* Pneumococci	89% (3rd dose)
* DTP	99% (1st dose); 95% (3rd dose)
* MMR	92% (1st dose); 79% (2nd dose)
* Polio	99% (1st dose); 86% (3rd dose)
* Rotaviruses	88%
** Varicella	123,743 (number of vaccinated individuals)
** Meningococci	135,679 (number of vaccinated individuals)
** Influenza	1,269,438 (number of vaccinated individuals)
** Hepatitis A	51,860 (number of vaccinated individuals)
TBE	102,147 (number of vaccinated individuals)
* Hepatitis B	93% (1st dose); 90% (3rd dose)
** RSV	30 (number of vaccinated individuals)

* World Health Organisation. WHO Immunisation Data Portal; ** National Institute of Public Health NIH—National Research Institute (NIPH NIH–NRI). Infectious Diseases and Vaccinations in Poland in 2023. DTP—Diphtheria–tetanus–pertussis; Hib—*Haemophilus influenzae* type b; MMR—Measles–mumps–rubella; TBE—Tick-borne encephalitis; RSV—Respiratory syncytial virus.

**Table 2:** Mandatory childhood vaccination schedules across EU/EEA countries in 2026, according to the ECDC Vaccine Scheduler [52].

Country	Number of Mandatory Vaccinations	Tuberculosis	DTP	Polio	Hib	Pneumococci	MMR	Varicella	Hepatitis B	Meningococci	Rotaviruses
Bulgaria	7	+	+	+	+	+	+	+			
Croatia	7	+	+	+	+	+	+		+		
Czechia	6	+	+	+	+		+		+		
France	7		+	+	+	+	+		+	+	
Latvia	9	+	+	+	+	+	+	+	+		+
Poland	9	+	+	+	+	+	+	+	+		+
Slovakia	6		+	+	+	+	+		+		
Hungary	8	+	+	+	+	+	+	+	+		
Italy	6		+	+	+		+	+	+		

+: the administration of a vaccine.

**Table 3:** DTP1 and DTP3 vaccination coverage rates and corresponding DTP dropout rates in EU/EEA countries without mandatory vaccination schedules, according to the 2024 WUENIC report [36].

Country	DTP1	DTP3	Vaccination Dropout Rate
Austria	95%	85%	10.5%
Belgium *	98%	97%	1%
Cyprus	99%	94%	5%
Denmark	97%	96%	1%
Estonia	82%	81%	1.2%
Finland	98%	91%	7.1%
Germany	97%	89%	8.2%
Greece	98%	95%	3.1%
Iceland	98%	94%	4.1%
Ireland	92%	92%	0%
Liechtenstein **	-	-	-
Lithuania	92%	89%	3.3%
Luxembourg	99%	99%	0%
Netherlands	91%	91%	0%
Norway	99%	97%	2%
Romania	85%	66%	22.4%
Spain	98%	94%	4.1%
Sweden	97%	96%	1%

* In Belgium, only vaccination against polio is mandatory. ** Data for Liechtenstein are not available. The DTP dropout rate was defined as the percentage difference between the proportion of children who received the first dose of the vaccine (DTP1) and the proportion of children who received the third dose (DTP3), expressed relative to the proportion of children who received the first dose (DTP1) [29].

**Table 4:** Reported vaccination coverage in EU/EEA countries without multi-antigen mandatory childhood vaccination schedules, according to the WUENIC report [36], European Commission Eurostat 2025 influenza statistics [53]. The table presents the most recent data available for each country by the review cut-off date. Reference periods differ among countries and indicators; therefore, the values do not constitute a single cross-sectional comparison and should not be interpreted as a standardized country ranking.

Country	Influenza *	Hib **	*S. pneumoniae* Children **	Measles **	Polio **	Rubella **	Hepatitis B **	Rotaviruses **
Austria	-	85%	-	90% (1st dose); 84% (2nd dose)	95% (1st dose); 85% (3rd dose)	90%	85% (3rd dose)	61% (final dose)
Belgium	Approx. 55%	97%	94% (3rd dose)	96% (1st dose); 82% (2nd dose)	99% (1st dose); 98% (3rd dose)	96%	97% (3rd dose)	86% (final dose)
Cyprus	Approx. 30%	94%	82% (3rd dose)	99% (1st dose); 97% (2nd dose)	99% (1st dose); 94% (3rd dose)	99% (1st dose)	94% (3rd dose)	-
Denmark	77.5%	96% (3rd dose)	96% (3rd dose)	94% (1st dose); 93% (2nd dose)	97% (1st dose); 96% (3rd dose)	94% (1st dose)	-	-
Estonia	Approx. 28%	81% (3rd dose)	-	83% (1st dose); 74% (2nd dose)	82% (1st dose); 81% (3rd dose)	83% (1st dose)	81% (3rd dose)	75% (final dose)
Finland	Approx. 61%	91% (3rd dose)	91% (3rd dose)	95% (1st dose); 91% (2nd dose)	98% (1st dose); 91% (3rd dose)	95% (1st dose)	-	85% (final dose)
Germany	Approx. 40%	88% (3rd dose)	75% (3rd dose)	96% (1st dose); 92% (2nd dose)	97% (1st dose); 88% (3rd dose)	96% (1st dose)	85% (3rd dose)	68% (final dose)
Greece	Approx. 64%	95% (3rd dose)	90% (3rd dose)	96% (1st dose); 71% (2nd dose)	98% (1st dose); 95% (3rd dose)	96% (1st dose)	92% (3rd dose)	20% (final dose)
Iceland	Approx. 52%	94% (3rd dose)	93% (3rd dose)	97% (1st dose); 98% (2nd dose)	98% (1st dose); 94% (3rd dose)	97% (1st dose)	-	-
Ireland	75.7%	92% (3rd dose)	84% (3rd dose)	90% (1st dose); 89% (2nd dose)	93% (1st dose); 92% (3rd dose)	90% (1st dose)	92% (3rd dose)	88% (final dose)
Liechtenstein	Approx. 23%	-	-	-	-	-	-	-
Lithuania	Approx. 24%	89% (3rd dose)	87% (3rd dose)	86% (1st dose); 85% (2nd dose)	92% (1st dose); 89% (3rd dose)	86% (1st dose)	93% (1st dose); 87% (3rd dose)	77% (final dose)
Luxembourg	Approx. 42%	99% (3rd dose)	97% (3rd dose)	99% (1st dose); 94% (2nd dose)	99% (1st dose); 99% (3rd dose)	99% (1st dose)	98% (3rd dose)	89% (final dose)
Netherlands	Approx. 66%	89% (3rd dose)	88% (3rd dose)	89% (1st dose); 81% (2nd dose)	91% (1st dose); 91% (3rd dose)	89% (1st dose)	89% (3rd dose)	-
Norway	Approx. 64%	97% (3rd dose)	95% (3rd dose)	96% (1st dose); 94% (2nd dose)	99% (1st dose); 97% (3rd dose)	96% (1st dose)	96% (3rd dose)	95% (final dose)
Portugal	71.8%	99% (3rd dose)	98% (3rd dose)	99% (1st dose); 96% (2nd dose)	99% (1st dose); 99% (3rd dose)	99% (1st dose)	99% (3rd dose)	-
Romania	Approx. 23%	66% (3rd dose)	66% (3rd dose)	66% (1st dose); 68% (2nd dose)	85% (1st dose); 66% (3rd dose)	66% (1st dose)	79% (1st dose); 66% (3rd dose)	-
Spain	Approx. 68%	94% (3rd dose)	92% (3rd dose)	97% (1st dose); 92% (2nd dose)	98% (1st dose); 94% (3rd dose)	97% (1st dose)	94% (3rd dose)	70% (final dose)
Sweden	Approx. 63%	95% (3rd dose)	94% (3rd dose)	93% (1st dose); 92% (2nd dose)	97% (1st dose); 95% (3rd dose)	93% (1st dose)	95% (3rd dose)	85% (final dose)

* European Commission Eurostat 2025 [53] influenza statistics for individuals aged 65 years or older (influenza vaccination rates in 2023). ** World Health Organisation. WHO Immunisation Data Portal 2024 [36]. Belgium was included because only vaccination against poliomyelitis is mandatory.

**Table 5:** Influenza vaccine types recommended for adults in selected European countries [77,78,79,80,81,82,83,84,85,86,87,88,89,90,91,92,93,94,95,96,97,98,99,100,101,102,103,104,105,106,107].

Country	Vaccine Type	Reference	Reference
Austria	IIV3 aIIV3 IIV3-HD	[77]	[77]
Belgium	IIV3 aIIV3 IIV3-HD	[78,79]	[78,79]
Bulgaria	IIV3 IIV4	[80,81]	[80,81]
Croatia	IIV3	[82]	[82]
Cyprus	IIV4 aIIV4	[83,84]	[83,84]
Czechia	IIV3 IIV3-HD	[85]	[85]
Denmark	IIV IIV3 aIIV3	[86,87]	[86,87]
Estonia	IIV4	[88]	[88]
Finland	IIV3	[89]	[89]
France	IIV4	[21]	[21]
Germany	IIV3 aIIV3 IIV3-HD	[90]	[90]
Greece	IIV3 aIIV3 IIV3-HD	[91]	[91]
Hungary	IIV3	[92]	[92]
Ireland	IIV3	[93]	[93]
Italy	IIV3 aIIV3 IIV3-HD	[94]	[94]
Latvia	LAIV IIV3-HD IIV4	[95]	[95]
Lithuania	LAIV IIV3-HD IIV4	[96]	[96]
Luxembourg	IIV3 IIV3-HD	[97]	[97]
Malta	IIV4	[98]	[98]
Netherlands	IIV	[99]	[99]
Poland	IIV4 IIV4-HD	[100]	[100]
Portugal	IIV4 IIV4-HD	[101]	[101]
Romania	LAIV IIV3 IIV4 IIV4-HD	[102]	[102]
Slovakia	IIV	[103]	[103]
Slovenia	IIV4	[104]	[104]
Spain	LAIV IIV3 aIIV3 IIV3-HD	[105]	[105]
Sweden	IIV3 IIV4	[106]	[106]

IIV3—trivalent inactivated influenza vaccine; aIIV3—adjuvanted trivalent inactivated influenza vaccine; IIV3-HD—high-dose trivalent inactivated influenza vaccine; IIV4—quadrivalent inactivated influenza vaccine; aIIV4—adjuvanted quadrivalent inactivated influenza vaccine; IIV4-HD—high-dose quadrivalent inactivated influenza vaccine; LAIV—live attenuated influenza vaccine; IIV—inactivated influenza vaccine.

**Table 6:** Pneumococcal vaccine types recommended for adults in selected European countries [77,80,82,83,85,88,90,92,93,97,99,100,101,102,103,104,105,106,107,116,117,118,119,120,121,122,123,124,125,126].

Country	Vaccine Type	Reference
Austria	PCV21	[77]
Belgium	PCV20 PCV21	[116,117]
Bulgaria	PCV13	[80]
Croatia	PCV15 + PPSV23	[82]
Cyprus	PCV20	[83]
Czechia	PCV20 PCV21	[85]
Denmark	No recommendations for adult vaccination	[118]
Estonia	No recommendations for adult vaccination	[88]
Finland	PCV13	[119]
France	PCV20 PCV21	[90]
Germany	PCV20	[120]
Greece	PCV20	[91]
Hungary	PCV20 PCV15 + PPSV23	[92]
Ireland	PPSV23	[121]
Italy	PCV13 + PPSV23	[122]
Latvia	PCV13 PCV15 PCV20 PCV21	[123]
Lithuania	PCV20	[96]
Luxembourg	PCV PCV20	[21]
Malta	PCV20	[98]
Netherlands	PCV20	[99]
Poland	PCV13 + PPSV23	[100]
Portugal	PPSV23 PCV20 + PPSV23	[101]
Romania	PCV13 PCV15 PCV20 PPSV23 PCV13 + PPSV23 PCV15 + PPSV23	[124]
Slovakia	PCV	[103]
Slovenia	PCV20	[104]
Spain	PCV PCV + PPSV	[105]
Sweden	PCV20 PCV21	[106]

PCV21—21-valent pneumococcal conjugate vaccine; PCV20—20-valent pneumococcal conjugate vaccine; PCV13—13-valent pneumococcal conjugate vaccine; PCV15—15-valent pneumococcal conjugate vaccine; PPSV23—23-valent pneumococcal polysaccharide vaccine; PCV—pneumococcal conjugate vaccine; PPSV—pneumococcal polysaccharide vaccine.

## Data Availability

No new data were created or analyzed in this study. Data sharing is not applicable to this article.

## Abbreviations

ACIP, Advisory Committee on Immunisation Practices; aIIV3, adjuvanted inactivated influenza vaccine, trivalent; aIIV4, adjuvanted inactivated influenza vaccine, quadrivalent; CIN3, cervical intraepithelial neoplasia grade 3; COVER, Cover of Vaccination Evaluated Rapidly; CSS, Conseil Supérieur de la Santé (Superior Health Council); DTP, diphtheria, tetanus, and pertussis; ECDC, European Centre for Disease Prevention and Control; EIW, European Immunisation Week; EMA, European Medicines Agency; EPI, Expanded Programme on Immunisation; EU/EEA, European Union/European Economic Area; GPs, general practitioners; HA, recombinant hemagglutinin; HGR, Hoge Gezondheidsraad (Superior Health Council); Hib, Haemophilus influenzae type b vaccine; HPV, human papillomavirus; IIV, inactivated influenza vaccine; IIV3, inactivated influenza vaccine, trivalent; IIV3-HD, high-dose inactivated influenza vaccine, trivalent; IIV4, inactivated influenza vaccine, quadrivalent; IIV4-HD, high-dose inactivated influenza vaccine, quadrivalent; IPD, invasive pneumococcal disease; LAIV, live attenuated influenza vaccine; MMR, measles–mumps–rubella; NHS, National Health Service; NIH, National Institute of Public Health (NIPH–NIH); PCV, pneumococcal conjugate vaccine; PCV13, 13-valent pneumococcal conjugate vaccine; PCV15, 15-valent pneumococcal conjugate vaccine; PCV20, 20-valent pneumococcal conjugate vaccine; PCV21, 21-valent pneumococcal conjugate vaccine; PPSV, pneumococcal polysaccharide vaccine; PPSV23, 23-valent pneumococcal polysaccharide vaccine; PSO, Program Szczepień Ochronnych (Vaccination Schedule); RSV, respiratory syncytial virus; STIKO, Ständige Impfkommission (Standing Committee on Vaccination); TBE, tick-borne encephalitis; UKHSA, UK Health Security Agency; UNICEF, United Nations Children’s Fund; WHO, World Health Organisation; WUENIC, WHO/UNICEF Estimates of National Immunisation Coverage.
